# Development and In Vivo Application of a Water-Soluble Anticancer Copper Ionophore System Using a Temperature-Sensitive Liposome Formulation

**DOI:** 10.3390/pharmaceutics12050466

**Published:** 2020-05-20

**Authors:** Anikó Gaál, Tamás M. Garay, Ildikó Horváth, Domokos Máthé, Dávid Szöllősi, Dániel S. Veres, Jeremiah Mbuotidem, Tibor Kovács, József Tóvári, Ralf Bergmann, Christina Streli, Gergely Szakács, Judith Mihály, Zoltán Varga, Norbert Szoboszlai

**Affiliations:** 1 Biological Nanochemistry Research Group, Institute of Materials and Environmental Chemistry, Research Centre for Natural Sciences, Magyar Tudósok Körútja 2, H-1117 Budapest, Hungary; gaal.aniko@ttk.mta.hu (A.G.); mihaly.judith@ttk.mta.hu (J.M.); 2 Faculty of Information Technology and Bionics, Pázmány Péter Catholic University, H-1083 Budapest, Práter utca 50/a, Hungary; 3 1st Department of Internal Medicine and Oncology, Semmelweis University, H-1083 Budapest, Hungary; 4 Department of Biophysics and Radiation Biology, Semmelweis University, H-1094 Budapest, Hungary; panpills@gmail.com (I.H.); domokos.mathe@cromedresearch.com (D.M.); szollosi.sote@gmail.com (D.S.); daniel.s.veres@gmail.com (D.S.V.); r.bergmann@hzdr.de (R.B.); 5 CROmed Translational Research Centers Ltd., H-1047 Budapest, Hungary; 6 Institute of Translational Medicine, Semmelweis University, H-1094 Budapest, Hungary; thomas_jeremiah.mbuotidem_ex@med.semmelweis-univ.hu; 7 Institute of Radiochemistry and Radioecology, University of Pannonia, H-8200 Veszprém, Hungary; kt@almos.uni-pannon.hu; 8 Department of Experimental Pharmacology, National Institute of Oncology, H-1122 Budapest, Hungary; tozsi@oncol.hu; 9 Helmholz-Zentrum Dresden-Rossendorf, Institute of Radiopharmaceutical Cancer Research, D-01328 Dresden, Germany; 10 Institute of Atomic and Subatomic Physics, Atominstitut, TU Wien, A-1020 Vienna, Stadionallee 2, Austria; christina.streli@tuwien.ac.at; 11 Institute of Enzymology, Research Centre for Natural Sciences, Magyar tudósok körútja 2, H-1117 Budapest, Hungary; szakacs.gergely@ttk.mta.hu; 12 Institute of Cancer Research, Medical University Vienna, A-1090 Vienna, Austria; 13 Laboratory for Environmental Chemistry and Bioanalytics, Institute of Chemistry, Eötvös Loránd University, H-1117 Budapest, Pázmány Péter Stny. 1/A, Hungary

**Keywords:** neocuproine, themosensitive liposomal formulation, mild hyperthermia, copper nanotoxin, MRPS, in vivo antitumor effect

## Abstract

Liposomes containing copper and the copper ionophore neocuproine were prepared and characterized for in vitro and in vivo anticancer activity. Thermosensitive PEGylated liposomes were prepared with different molar ratios of 1,2-dipalmitoyl-sn-glycero-3-phosphatidylcholine (DPPC) and hydrogenated soybean phosphatidylcholine (HSPC) in the presence of copper(II) ions. Optimal, temperature dependent drug release was obtained at 70:30 DPPC to HSPC weight ratio. Neocuproine (applied at 0.2 mol to 1 mol phospholipid) was encapsulated through a pH gradient while using unbuffered solution at pH 4.5 inside the liposomes, and 100 mM HEPES buffer pH 7.8 outside the liposomes. Copper ions were present in excess, yielding 0.5 mM copper-(neocuproine)_2_ complex and 0.5 mM free copper. Pre-heating to 45 °C increased the toxicity of the heat-sensitive liposomes in short-term in vitro experiments, whereas at 72 h all investigated liposomes exhibited similar in vitro toxicity to the copper(II)-neocuproine complex (1:1 ratio). Thermosensitive liposomes were found to be more effective in reducing tumor growth in BALB/c mice engrafted with C26 cancer cells, regardless of the mild hyperthermic treatment. Copper uptake of the tumor was verified by PET/CT imaging following treatment with [^64^Cu]Cu-neocuproine liposomes. Taken together, our results demonstrate the feasibility of targeting a copper nanotoxin that was encapsulated in thermosensitive liposomes containing an excess of copper.

## 1. Introduction

Cancer is the second most frequent cause of death [1,2]. Treatment of advanced stage diseases is still a major clinical problem despite the availability of a broad range of therapeutic agents with different mechanisms of anticancer activity. The discovery of cisplatin ([*cis*-diammine-dichloroplatinum(II) complex]) [3] laid the foundation for the use of metal-based compounds in the treatment of several cancers, including testicular cancer, ovarian cancer, or breast cancer. Second and third generation platinum (Pt) drugs were developed in order to reduce the dose-limiting toxicity of cisplatin and overcome the intrinsic or acquired cisplatin resistance of cancer cells [4]. The development of further Pt-based compounds has been the subject of intensive research [5,6,7]. In parallel, alternative metal-based complexes are also considered. Copper (Cu) chelating compounds are primary candidates for development [8,9,10], given the strong in vitro toxicity of copper complexes. Common examples of copper chelating ligands include derivates of dithiocarbamate [11], 8-hydroxyquinoline [12], thiosemicarbazone [13,14], and phenantroline structures [15]. These ligands form stable and highly toxic copper complexes with persistent toxicity, even in multidrug resistant cell lines.

The essentiality of copper (Cu) is partially linked to its involvement in basic electron transfer processes through the reduction of Cu(II) to Cu(I)) [16]. However, the same one electron exchange reaction also generates free radicals that can be harmful to cells [17]. Cells efficiently protect their intracellular milieu by hiding copper from cellular constituents, thereby preventing the accumulation of free ionic copper. Copper chelator structures can circumvent these defensive processes. The exact mechanism by which copper increases the toxicity of certain chelator compounds is not known [13,15]. The toxicity of copper chelating ionophores is usually accompanied by the increased cellular accumulation of copper [18], which is primarily responsible for cell death [15]. This is in contrast to the mechanism of toxicity of another group of chelators including tetrathiomolybdate, which kill cells by copper depletion [10,19]. Compounds belonging to this second group do not cross cell membranes and are ionic in their chemical nature.

1,10-phenantrolines are typical copper ionofores, and copper complexes of 1,10-phenantrolines show antineoplastic properties [20]. Both the Cu(I) and Cu(II) complexes of 2,9-dimethyl-1,10-phenantroline (neocuproine) have been shown to be potent cytotoxic agents against different cell lines [21,22], and their activity was potentiated by adding Cu(II) ions to the cell culture medium [23,24,25]. The lipophilic Cu(I)-specific chelator neocuproine is frequently used as an inhibitor of Cu-mediated damage in biological systems [26].

Drug delivery systems (DDSs), such as liposomes, can be utilized to formulate Cu complexes. Liposomes can be manufactured in various sizes, depending on the desired in vivo behavior, and they can encapsulate a wide range of molecules with hydrophilic, amphiphilic, or lipophilic characteristics. Liposomes with an average diameter of 100 nm have been found to remain in circulation for a significantly longer period than liposomes of a larger size. Most DDSs target malignant tissues rather than healthy organs, even in the absence of a targeting ligand, due to the Enhanced Permeability and Retention (EPR) effect [27]. The EPR effect is due to the poor lymphatic drainage and the defective blood vasculature of tumor tissues, which results in the extravasation of nanoparticles into the tumor area.

Although many Cu complexes have been synthesized as anticancer agents [9,28], their in vivo efficacy remains questionable, and there are currently no promising copper complexes under clinical investigation. The main challenge in the clinical development is the high toxicity and low selectivity of copper complexes. Both selective targeting and toxicity reduction can be achieved by formulation strategies coupled with a homing (targeting) system. Liposomal encapsulation provides a solution to increase the concentration of cytotoxic copper complexes in the tumor, while decreasing the blood concentration and, thus, reducing general toxicity. Active targeting can be achieved by using thermosensitive liposomes and mild hyperthermia to further reduce unwanted toxicity and to enhance drug delivery [29].

To date, only a few liposomal formulations containing Cu alone or Cu complex precipitates have been reported [30,31,32]. Different chelators can be loaded into copper-containing systems at different temperatures [33]. Liposomal formulation was most successful in the case of copper complexes forming precipitates, including clioquinol, quercetin and CX-5461 [33]. The liposomal formulation of the copper complex of diethyldithiocarbamate showed promising in vivo results in a platinum-resistant A2780-CP ovarian xenograft model [34].

Here, we describe the development and biological characterization of a drug delivery system consisting of liposomes loaded with excess copper and the water-soluble active ingredient neocuproine.

## 2. Materials and Methods

### 2.1. Chemicals

Throughout the experiments, deionized Milli-Q (Millipore, Molsheim, France) water with a resistivity of 18.2 MΩ·cm was used. All of the chemicals were of analytical grade, if not stated otherwise. Copper(II) sulfate, neocuproine (2,9-Dimethyl-1,10-phenanthroline) hydrochloric acid salt, 4-(2-Hydroxyethyl)piperazine-1-ethanesulfonic acid sodium salt (HEPES sodium salt), ascorbic acid, bovine serum albumin (BSA), and hydrochloric acid were purchased from Sigma Aldrich Ltd. (Budapest, Hungary); NaCl 0.9% was obtained from B. Braun (Budapest, Hungary); chloroform and methanol were purchased from Reanal (Budapest, Hungary). Synthetic high-purity 1,2-dipalmitoyl-sn-glycero-3-phosphatidylcholine (DPPC), hydrogenated soybean phosphatidylcholine (HSPC), and 1,2-distearoyl-sn-glycero-3-phosphoethanolamine-*N*-[amino(polyethylene glycol)-2000] (ammonium salt) (DSPE-PEG 2000) were obtained from Avanti Polar Lipids (Alabaster, AL, USA) or Sigma Aldrich. The chemicals were used without further purification. Disposable PD-10 and PD MidiTrap G-25 desalting columns were obtained from GE Healthcare Life Sciences (Budapest, Hungary). Concentrated nitric acid (65% *w*/*w*) and H_2_O_2_ (30% *w*/*w*) of Suprapur quality needed for the sample preparation of cell lines for Total-Reflection X-ray Fluorescence (TXRF) measurement were supplied by Merck (Darmstadt, Germany).

### 2.2. Preparation of Drug-Loaded Liposomes

Thermo-sensitive and thermo-resistant liposomes were prepared by the lipid film hydration and extrusion method. The lipid mixtures containing DPPC, HSPC and DSPE-PEG2000 in the weight ratios summarized in Table 1 were dissolved in chloroform and dried to a thin lipid film under a stream of N_2_ gas, followed by incubation overnight under vacuum to remove residual solvent.

Next, 100 mM (or 10 mM, 300 mM for sample LIPO1) of CuSO_4_ solution was used to hydrate the lipid films to gain a total lipid concentration of 35 mg/3 mL, while mixing with a magnetic stirrer in a 60 °C water bath for 30 min. The resulting multilamellar vesicle (MLV) suspension was subjected to five cycles of freeze-and-thaw (5 min. each, freezing in liquid nitrogen, and thawing at 60 °C) before being extruded 13 times at 60 °C through a 100 nm polycarbonate membrane filter (Whatman, Springfield Mill, UK) via a LIPEX^®^ Extruder (LIPEX^®^ Biomembranes, Burnaby, BC, Canada) to generate large unilamellar vesicles (LUVs) encapsulating the Cu salt. Residual un-encapsulated Cu was removed by size exclusion chromatography by passing 2.5 mL liposome suspension through a desalting PD-10 column and eluting with 3.5 mL 100 mM HEPES buffer (pH = 7.8), prepared from Hepes-Na salt, and setting the pH by hydrochloric acid. The pH of the buffer was manually adjusted using S20 SevenEasy™ pH-meter (Mettler Toledo, Budapest, Hungary). The resulting liposomal formulations contained the indicated copper ion solution (typically an unbuffered solution that was at pH 4.5) inside, and the HEPES buffer pH 7.8 outside [35]. After the separation process, 0.1 M neocuproine hydrochloric acid (dissolved in MQ water) was added in 0.2:1 mol drug-to-mol phospholipid ratio and incubated overnight at room temperature for loading of the neocuproine into the liposome. The unencapsulated neocuproine, or copper-neocuproine complex was removed by loading 1 mL sample on a G-25 Midi-Trap column and eluting the purified liposome fraction with 1.5 mL sterile 0.9% NaCl solution. Dynamic Light Scattering (DLS), Microfluidic Resistive Pulse Sensing (MRPS), Differential Scanning Calorimetry (DSC), IR spectroscopy, and UV-VIS spectrophotometry were used to characterize the prepared liposomes. For the Cu determination, the liposomes were dissolved in methanol and diluted 10-fold by Milli-Q water. The copper content of the samples was measured by the TXRF method (see Section 2.12) using 1500 ng Ga (Gallium standard solution) as an internal standard. The neocuproine content and encapsulation efficacy of the neocuproine was measured by spectrophotometry. An aliquot (20 μL) was dissolved in 50 μL methanol and then diluted with deionized water to 100 µL to release encapsulated Cu and neocuproine from liposomes. The complex was reduced with ascorbic acid and the absorption of the samples was measured at 450 nm. Calibrating solutions were prepared with the Cu-neocuproine ratio 1:1. The encapsulation efficacy was calculated as a proportion of the neocuproine administered and recovered after column filtration. Neocuproine free liposomes were also prepared for IR study, apart from these, all of the liposomes in the following are drug loaded and PEGylated.

### 2.3. Dynamic Light Scattering (DLS)

The mean particle diameter and size distribution function of the samples during the preparation procedure were determined using dynamic light scattering (DLS) at 20 °C. DLS measurements performed on a W130i apparatus (Avid Nano Ltd., High Wycombe, UK) and using a low-volume disposable cuvette (UVette, Eppendorf Austria GmbH, Wien, Austria), which was equipped with a diode laser (λ = 660 nm) and a side scatter detector at fixed angle of 90°. For these experiments, the samples were diluted 10-fold with 0.9% NaCl solution, data evaluation was performed with iSIZE 3.0 software (Avid-Nano), utilizing the CONTIN algorithm.

### 2.4. Microfluidic Resistive Pulse Sensing (MRPS)

Microfluidic resistive pulse sensing (MRPS) is the nanoscale implementation of the coulter principle in a microfluidic cartridge [36,37,38,39]. The MRPS measurements were performed with a nCS1 instrument (Spectradyne LLC, Torrance, CA, USA). The samples were diluted 500-fold with BSA solution at 1 mg/mL in 0.9% NaCl, filtered through a VivaSpin 500, 300 kDa MWCO membrane filter (Sartorius, Budapest, Hungary), according to the manufacturer’s instructions. All of the measurements were performed using factory calibrated TS-300 cartridges with a measurement range from 50 nm to 300 nm.

### 2.5. IR Spectroscopy

The IR measurements were carried out using a Varian FTS-2000 (Scimitar Series) FTIR spectrometer (Varian Inc., Agilent, Santa Clara, CA, USA) that was equipped with a ‘Golden Gate’ single reflection diamond attenuated total reflection (ATR) accessory (Specac Ltd., Orpington, UK). 3 µL of preformed copper-neocuproine complex (1:1) or liposome samples were spread on the surface of the diamond ATR element and a thin dry film was obtained by slowly evaporation of the buffer solvent under ambient conditions. A spectral resolution of 2 cm^−1^ and co-addition of 64 individual spectrum were adjusted. ATR correction and buffer background subtraction were applied for all spectra. Spectral manipulations, including also curve-fitting procedure, were performed by the GRAMS/32 software package. For curve-fitting, band positions were estimated while using the second derivative, and band shapes were approximated by Gauss functions until the minimum of χ^2^ was reached.

### 2.6. Differential Scanning Calorimetry (DSC)

DSC was used to characterize thermotropic phase transitions of liposome samples. The measurements were performed on a μDSC 3 EVO (Setaram, Caluire-et-Cuire, France) instrument in the 20 °C to 60 °C temperature range with 0.2 °C/min. heating rate using 300–400 mg of the liposome samples.

### 2.7. Stability of the Liposomes

In order to test the stability of the liposomes 200 µL samples were centrifuged through Zeba™ Spin Desalting Columns, 40 K MWCO, 0.5 mL (Thermo Fisher, Waltham, MA, USA) after previous washing procedures according to the instructions of the manufacturer. The release of the copper-neocuproine complex from the liposomes was tested at different temperatures (temperature dependence release, each liposome was heated for 10 min by a Biosan (Biosan, Latvia) CH-100 heating block and at different timepoints after the preparation (time dependence of the stability). Absorbance was spectrophotometrically measured at 450 nm (maximal absorbance of Cu(I)-NEO complex) while using an EnSpire microplate reader (Perkin Elmer, Waltham, MA, USA). The data were normalized to unheated or freshly prepared liposomes.

### 2.8. Size Exclusion Chromatography (SEC)

100 µL liposome sample was incubated with 900 µL completed DMEM medium (containing 10% FBS) for four hours at 37 °C, 5% CO_2_ modeling cell culture conditions. 10 µL of incubated sample was injected into a Jasco HPLC system (Jasco, Tokyo, Japan) consisting of a PU-2089 pump with an UV-2075 UV/Vis detector and using a Tricorn 5/100 glass columns (GE Healthcare Bio-Sciences AB, Uppsala, Sweden), filled with Sepharose CL-2B (GE Healthcare Bio-Sciences AB) were used, and the eluent was PBS with a flow rate of 0.5 mL/min. The UV-Vis chromatograms were collected at 450 nm wavelength corresponding to the absorption maximum of Cu(I)-neocuproine complex.

### 2.9. Cell Lines

The following cancer cell lines were used: HT-29 human colon adenocarcinoma and C26 mouse colon carcinoma, both obtained from ATCC (LGC Standards GmbH, Wesel, Germany) and cultured in RPMI and DMEM, respectively. RPMI and DMEM (Sigma Aldrich, Budapest, Hungary) were supplemented with 10% FBS (fetal bovine serum, Gibco, Thermo Fisher), 5 mmol/L glutamine, and 50 unit/mL penicillin and streptomycin (Life Technologies, Carlsbad, CA, USA). The cell cultures were kept in a humidified incubator at 37 °C and in 5% CO_2_ atmosphere. Washing steps were executed by Dulbecco’s Phosphate Buffered Saline solution (DPBS, Gibco^TM^, Thermo Fischer Scientific, Waltham, MA, USA), cells were trypsinized by trypsin–EDTA solution (5.0 g/L porcine trypsin and 2.0 g/L EDTA·4 Na in 0.9% *v*/*v* sodium chloride solution purchased from Sigma Aldrich).

### 2.10. Cell Viability Assay

Briefly, the cells were seeded into 96-well tissue culture plates (Sarstedt, Newton, USA/Orange, Braine-l’Alleud, Belgium) at a density of 5000–20,000 cells per well/ 100 µL (depending on the treatment time). The cells were allowed to attach for 12 h. Test compounds were added to achieve the required final concentration in a final volume of 200 μL per well. The liposomes (all investigated liposomes was PEGylated and drug loaded see Section 2.2) were diluted 10× in the culture media and 100 µL was added to the cells in half dilutions. In the case where the liposomes have been heated, the diluted liposomes were heated in Eppendorf tubes while using a Biosan CH-100 heating block. After incubation (4 h, 24 h, 72 h), the supernatant was removed, cells were washed once with DPBS, and the viability was assessed by the PrestoBlue^®^ assay (Life Technologies, Carlsbad, CA, USA), according to the manufacturer’s instructions. The viability of the cells was spectrophotometrically measured (measuring fluorescence, excitation at 544 nm and emission at 590 nm) using an EnSpire microplate reader (Perkin Elmer). The data were normalized to untreated cells. The curves were fitted by Graph Pad Prism 8 software while using the sigmoidal dose–response (variable slope) model. Curve fit statistics were used to determine IC_50_ values.

### 2.11. Sample Preparation for Determination of Intracellular Cu Levels

The cells were seeded into six-well culture plates (10^6^ cells/well) in 2 mL media. Cells were incubated overnight, and the medium was changed to 2 mL FBS-free medium before the treatment. The cells were treated with different volumes (2–12 µL) of the liposomes. After a four-hour-long incubation, the cells were harvested by trypsinization. Cells were washed twice with 1 mL DPBS. The cell number was determined with a TC20™ Automated Cell Counter (Bio-Rad Laboratories, Budapest, Hungary) while using trypan blue. After the last centrifugation step, DPBS was completely removed and the cells were digested for 24 h at room temperature in 20 μL of 30% H_2_O_2_, 80 μL of 65% HNO_3_, and 15 μL of 10 μg/mL Ga. From the resulting solutions, 2 μL was pipetted on the quartz reflectors that were used for TXRF analysis.

### 2.12. Total-Reflection X-Ray Fluorescence (TXRF) Analysis

Cu content was determined by the TXRF method, as reported elsewhere [18,40,41], using an Atomika 8030C TXRF spectrometer (Atomika Instruments GmbH, Oberschleissheim, Munich, Germany). Gallium was used as an internal standard. The stock solution of 1000 mg/L Ga was purchased from Merck (Darmstadt, Germany). The Kα line used for the determination of Cu was at 8.047 keV.

### 2.13. In Vivo Anti-Tumor Efficacy of Drug-Containing Liposomes

2 × 10^6^ mouse colon carcinoma (C26) cells were injected into the left flank of 6–9 week-old male BALB/c mice from our specific pathogen-free colony subcutaneously (s.c.) in a volume of 0.2 mL serum-free media. Two weeks after injection (when the tumors were measurable), the mice were randomly and evenly divided into groups (10 mice/group). Treatment groups received 10 µL liposome/1g body weight intravenously (i.v.) on the first and eighth day of treatment. The concentration of the complex (Cu:neocuproine) was taken as 1 mM, resulting in an amount of 2.8 mg/kg of “active ingredient” calculated for the thermosensitive formulation. The dose of the encapsulated drug for the “thermoresitant” formulation was 2.6 mg/kg. The controls received equivalent volumes of sterile NaCl 0.9%. All of the animals were included in the analysis. Changes of the body weight were also determined throughout the study. No adverse events were observed during the experiment. The antitumor effects were registered by measuring tumor size with caliper twice a week. Tumor volume was calculated with the formula for a prolate ellipsoid (length × width2 × (π/6)). Eighteen days after the first treatment, the mice were euthanized, and the tumors were extracted after the experiments and then dried in an oven to a constant weight. The samples were weighed on an analytical balance. Statistical analysis was performed using Graph Pad Prism 8 software using One-Way ANOVA analyses. The animal experiments were carried out at the Department of Experimental Pharmacology, National Institute of Oncology, Budapest, Hungary, and the animal-model experiments were conducted following the standards and procedures approved by the Animal Care and Use Committee of the National Institute of Oncology, Budapest (license number: PEI/001/2574–6/2015). The Hungarian Animal Health and Animal Welfare Directorate approved all animal protocols according to the EU’s directives.

The tumors were heated to test the temperature dependent release of the liposomes in two treated animal groups. Animals were treated under anesthesia with desflurane (9% desflurane in 30% oxygen/air). Local mild hyperthermia (40–42 °C) was performed under anesthesia with a custom-made contact heating device based on direct heat conduction while using a metal rod that was connected to a temperature-controlled water bath. Intratumoral temperature was measured with optical sensors (Luxtron FOT Lab Kit, LumaSense Technologies, Inc. CA Santa Clara, CA, USA) and kept within 41–42 °C (±0.5 °C) for 20 min. The applied temperature (41–42 °C) is above the phase transition temperature of the thermosensitive liposome (HEAT SENS LIPO).

### 2.14. Radiolabeling of Liposomes with Cu-64 for In Vivo PET Imaging

1 mL extruded liposome sample containing HSPC (9 mg/3 mL), DPPC (21 mg/3 mL), and DSPE-PEG2000 (5 mg/3 mL) hydrated with 100 mM non-radioactive CuSO_4_ solution was first purified on a G-25 Midi-Trap column to remove non-entrapped Cu^2+^ ions. The purified liposome fraction was eluted with 1.5 mL sterile 0.9% NaCl solution and it was subsequently mixed with 200 μL (400 to 450 MBq) no carrier added [^64^Cu]CuCl_2_ (produced on the cyclotron TR-FLEX, Advanced Cyclotron Systems, Inc., Richmond, BC, Canada, at the Helmholtz-Zentrum Dresden-Rossendorf). After incubation at room temperature for 60 min, 0.425 mL 100 mM HEPES buffer and 24 μL 0.1 M neocuproine hydrochloric acid (dissolved in MQ water) stock solution was added to the liposome sample (thermosensitive formulation) and incubated overnight at room temperature for loading of the neocuproine into the liposomes. Finally, the sample was purified on a PD-10 column with sterile 0.9% NaCl solution resulting in 120 to 150 MBq radiolabeled liposomes in 2 mL volume (elution profile shown in Figure S1) with approx. 95 to 97% radiochemical purity (based on the desalting efficiency of the PD-10 column, according to the manufacturer).

### 2.15. Small Animal Imaging

Copper-64 (T_1/2_ = 12.7 h; β^+^, 0.653 MeV [17.8%]; β^−^, 0.579 MeV [38.4%]) has decay characteristics that allow for positron emission tomography (PET) imaging. NMRI-Foxn1^nu/nu^ mice (*n* = 3) with U251-HRE xenografts were used for the in vivo imaging experiments. [^64^Cu]Cu-neocuproine liposome suspensions (8.2 ± 0.8 MBq) were injected via the tail vein. One animal received local mild heat treatment following the liposome (thermosensitive formulation) injection, while the remaining two mice served as controls. PET/CT measurements were performed with a nanoScan^®^ PET/CT, (Mediso Ltd., Budapest, Hungary) with a system PET resolution of 0.9 mm and the detection limit of a focal signal of 0.073 mm^3^. All animals were anaesthetized with 1.5% isoflurane during imaging. PET data acquisition started one and four hours post-injection for the two control animals, and three hours post-injection for the animal that received mild hyperthermic treatment. The acquisition continued for 60 min. in list mode. A 5 ns coincidence window and 400–600 keV energy window was applied in 94.7 mm scan range. A three-dimensional (3D) expectation maximization (3D EM) PET reconstruction algorithm (Mediso Tera-TomoTM) was applied to produce PET images, including corrections for decay, attenuation and scatter, dead time, and randoms. The helical CT scans were acquired with 35 kV tube voltage, 300 ms exposure time, 1 mA tube current, 1:4 binning and 360 projections. The images were reconstructed with 0.12 mm isovoxel sizes. PET attenuation correction was based on the CT images. After 4 iterations the reconstruction resulted in images with 0.4 mm isovoxel size. The results of PET measurements were quantified in units of radioactivity measured per unit volume (MBq/mL). Image analysis was performed with VivoQuant software (inviCRO, Boston, MA, USA). Following registration of the PET and CT data, tumor and muscle volumes of interest (VOI) were selected by hand. The radioactivity concentrations of tumor VOI’s are presented as a ratio to the image-based whole-body radioactivity concentration. Animal whole-body volumes were determined in mm^3^ while using a semi-automatic image thresholding algorithm in VivoQuant based on CT densities and corrected with the PET volumes.

## 3. Results and Discussion

### 3.1. Optimization of the Liposomal Formulation

**Figure 1:**
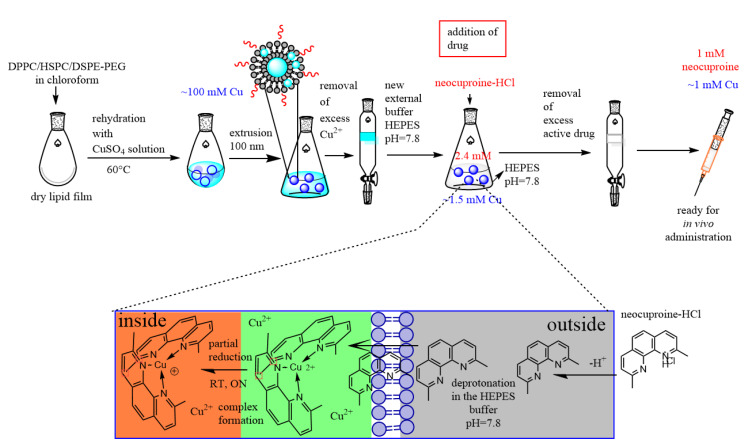
Schematic representation of the preparation of HEAT SENS LIPO via the thin film hydration method.

The main goal of this work was to characterize and test the in vivo anticancer activity of a novel copper-ionophore encapsulated in a temperature sensitive liposomal drug delivery system. The first step of the preparation of thermosensitive liposomes containing the copper-neocuproine complex was the formation of unilamellar liposomes in CuSO_4_ solution, which was followed by replacing the external solution with 100 mM Hepes-Na (pH 7.8) buffer by gel filtration (Figure 1). Next, neocuproine was added to the solution, which resulted in the formation of Cu(I)-neocuproine complex within the liposomes [42]. The Cu(II) concentration, the neocuproine-to-lipid ratio, and the lipid composition were optimized, and the resulting system was thoroughly characterized by DLS, MRPS, IR spectroscopy, and DSC, in order to achieve maximal loading efficiency while maintaining the stability and temperature sensitivity of the liposomal drug delivery system.

#### 3.1.1. Optimal Copper(II) Concentration

Different lipid-based copper containing PEGylated liposomes (using LIPO1, Table 1) were prepared by the hydration of the dry lipid film with CuSO_4_ solutions at 10 mM, 100 mM, 300 mM concentrations at 35 mg/mL lipid concentration. After extrusion through a polycarbonate membrane with nominal 100 nm pore size, the size distributions of the liposomal systems were measured by DLS (data not shown). The liposomes were then loaded with neocuproine while using a pH gradient (see Section 2.2). Loading with 300 mM CuSO_4_ resulted in unstable liposomes, as indicated by visible precipitation and an increase in the PDI of the size distribution measured by DLS, while loading with the lowest concentration of 10 mM CuSO_4_ loading resulted in significantly lower copper concentrations and reduced cytotoxicity, as measured after gel filtration with TXRF and the PrestoBlue assay, respectively (Supplementary Table S1). Hydration of the lipid films with 100 mM CuSO_4_ in MQ water solution was optimal, resulting a stable, monodisperse liposome system (D_avg_ = 103 ± 13 nm, PDI = 12%) with an encapsulated Cu(II) concentration of 1.0 ± 0.2 mM.

#### 3.1.2. Optimal Drug to Phospholipid Ratio

Neocuproine interacts with phospholipids due to its lipophilic character; therefore, the optimal drug-to-lipid concentration was investigated in the 0.2 mol–0.8 mol neocuproine to 1 mol lipid concentration range. Neocuproine stock solution was added to copper loaded (100 mM CuSO_4_ was used) HSPC lipid-based PEGylated liposomes (LIPO1 hereinafter HEAT RES LIPO and LIPO3 hereinafter HEAT SENS LIPO in Table 1) encapsulating 100 mM CuSO_4_ solution and suspended in 100 mM Hepes-Na buffer, and DLS measurements were performed after overnight incubation. In the final step of the preparation, non-encapsulated neocuproine was removed with gel filtration while using sterile 0.9% NaCl solution as eluent. Except for the conditions ensuring a 0.1 and 0.2 mol neocuproine-to-lipid molar ratio, all of the investigated neocuproine concentrations resulted in unstable liposome systems, as indicated by visible precipitation (Figure S2). The liposome that was prepared by 0.1 mol neocuproine-to-lipid molar ratio showed higher IC_50_ values (Table S2). Thus, liposomes loaded with 0.2 mol of neocuproine per 1 mol of phospholipid were selected for further investigations.

#### 3.1.3. Optimization of the Lipid Composition of Thermosensitive Liposomes

The main lipid component of HSPC-based liposomal formulations is 1,2-distearoyl-sn-glycero-3-phosphocholine (DSPC), which has a phase transition temperature at 55 °C [43,44]. The permeability of the lipid bilayer is known to be enhanced above this temperature, so using heat treatment lead to pronounced drug release. Mild hyperthermia applications require a phase transition temperature slightly above the physiological body temperature (39–40 °C); therefore, lipids with shorter fatty acid chains were added to the formulations, to lower the phase transition temperature. Liposomes with different compositions were prepared by varying the weight percentage of DPPC which has two-phase transition temperatures, the so-called pre-transition at 33 °C, and the main transition temperature at 41 °C and HSPC (Table 1). In our first experiments, the in vitro toxicity of the LIPO1-LIPO6 (copper and neocuproine loaded) liposome samples were measured with and without a 10 min. heat treatment at 45 °C, following incubation with the cells for four or 24 h. The results show that, without the heat treatment, liposomal formulations are devoid of any in vitro cytotoxic effect after four hours (Supplementary Table S3). A lack of toxicity after four hours of incubation indicates that the copper-neocuproine complex was successfully encapsulated, as the free form is already highly toxic within this relatively short time frame (Table S3). Heating the liposomes to 45 °C for 10 min. increased the cytotoxicity of all liposomal forms. As expected, HEAT RES LIPO formulations (LIPO1) containing exclusively HSPC were relative resistant to heat activation (IC_50_ changes from 33.1 µM to 27.5 µM), while the highest increase in toxicity was observed with LIPO6 (containing DPPC only, IC_50_ changes from >40 µM to 5.2 µM).

Short-term (4 h) cytotoxicity assays were repeated with heat treatment of the samples at increasing temperatures in order to find the ideal DPPC to HSPC (*w*/*w*%) ratio for mild hyperthermia applications (Table 2). LIPO1 and LIPO2 (containing no or 50% DPPC, respectively) were not cytotoxic, even after a heat treatment at 39 °C, whereas LIPO4, LIPO5, and LIPO6 (which contain 80%, 90% and 100% DPPC, respectively) proved to be cytotoxic following pre-heating of the samples to 38 °C. Based on these results, the optimal lipid content was determined to be 70 *w*/*w*% DPPC and 30 *w*/*w*% HSPC, resulting in a PEGylated liposomal formulation without a remarkable cytotoxic effect at 38 °C, and the release of the entrapped drug at 39 °C (Table 2.) In conclusion, LIPO3 (70 *w*/*w*% DPPC: 30 *w*/*w*% HSPC, denoted as HEAT SENS LIPO) was chosen for heat sensitive liposomal formulation strategies, because this formulation showed the desired drug-release behavior (no cytotoxicity at 37 °C and 38 °C; drug release at 39 °C).

#### 3.1.4. Characterization of the Optimized Liposomal Formulations

**Figure 2:**
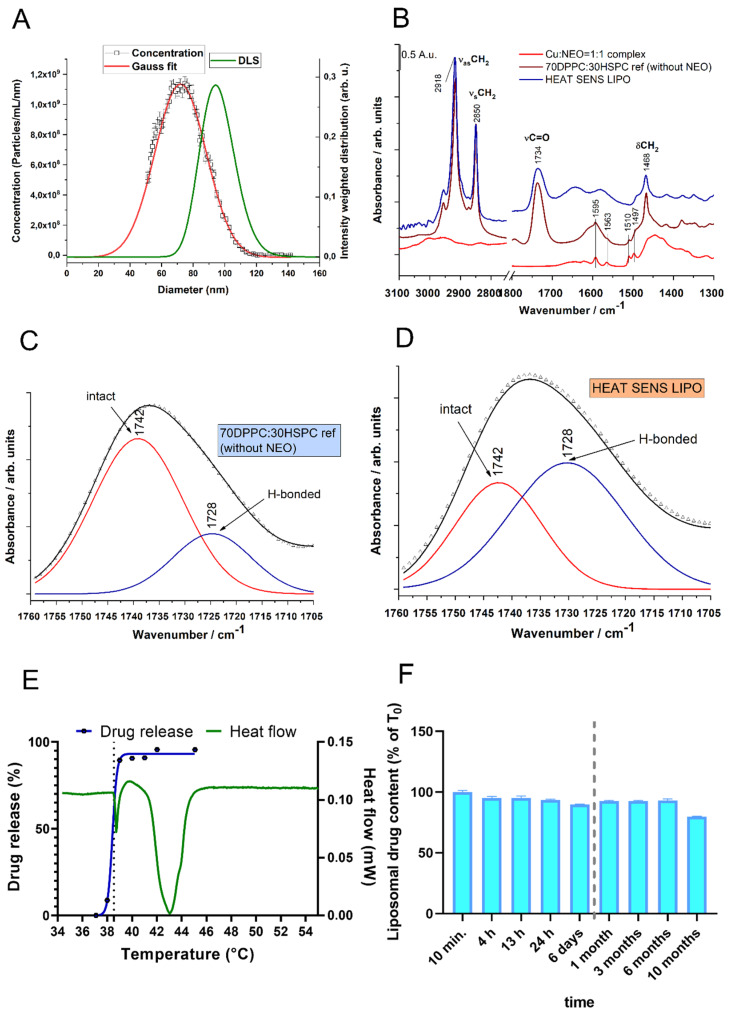
Characterization of the liposomal formulation HEAT SENS LIPO. (**A**) Size distributions obtained by DLS and MRPS. This figure denotes the evolution of particles with diameter sizes (nm) in the function of intensity (DLS) or concentration (MRPS) in the range of 0–160 nm (**B**) IR spectra of HEAT SENS LIPO without and with adding of neocuproine chelator compared to Cu(II)-neocuproine preformed complex IR spectra. (**C**) and **(D**) Part of IR spectra of Cu LIPO without adding neocuproine chelator (**C**) and HEAT SENS LIPO (**D**) samples. (**E**) Comparison between the heat flow (mW) measured by the DSC method for the HEAT SENS LIPO and its drug release profile. Tests were carried out with the normal 0.9% NaCl infusion. The pre-phase transition onset temperature for HEAT SENS LIPO release was relatively narrow to the expected, the measured value was 38.55 °C. (**F**) Stability of the liposomal formulation using gel filtration After 10 months, 80% of the active ingredient is still present in the liposome.

The mean hydrodynamic diameter was 103 nm ± 13 nm (PDI: 12%) and 97 nm ± 8 nm (PDI: 9%) for HEAT RES LIPO and HEAT SENS LIPO, respectively. The size distribution of HEAT SENS LIPO was further measured by MRPS (Figure 2A). The mean diameter was 72 ± 0.25 and SD was 32.5 ± 0.73, according to the best fit to the Gauss function (Figure 2A). The difference between the two measurements can be attributed to the thickness of hydration layer of the liposomes, because DLS measures the hydrodynamic diameter, whereas MRPS determines the diameter corresponding to the excluded volume [45]. MRPS also determined the particle concentration of 2.11 × 10^13^ ± 0.8% particle/mL.

TXRF/UV-Vis spectroscopy determined the copper/ neocuproine content of the HEAT SENS LIPO. The copper concentration of the HEAT SENS LIPO and HEAT RES LIPO was found to be similar (1 mM ± 0.05 mM). Neocuproine concentration was 1 mM ± 0.1mM, with an encapsulation efficacy of 64% for HEAT SENS LIPO, thus there is an excess of copper in the liposomes, since the complex ratio is 1: 2 for Cu:neocuproine. In the case of HEAT RES LIPO, the neucoproine concentration was slightly lower 0.9 mM ± 0.1 mM, with an encapsulation efficacy of 55%.

Infrared measurements with the copper-neocuproine complex, HEAT SENS LIPO before the addition of neocuproine and after the final gel purification step clearly indicate the presence of the complex in the final product (Figure 2B). Detailed spectral analysis of the C=O stretching vibration (1750–1720 cm^−1^) [46,47] of the liposome sample before the addition of neocuproine indicates that copper ions reduce the number of H-bonds around the carbonyl groups of the phospholipids, presumably because of the binding of the metal ions to the hydrophobic–polar interface of the lipid bilayer (Figure 2C). After the addition of neocuproine, the number of H-bonds around the carbonyl groups is significantly increased (Figure 2D), indicating the formation of the copper-neocuproine complex.

DSC measurement was carried out in order to reveal the thermotropic behavior of the optimized liposomal system (HEAT SENS LIPO). In parallel, drug release was monitored in the 37 °C to 45 °C temperature range by UV-Vis spectroscopy following separation of the liposomes from the released copper-neocuproine complex by gel filtration (Figure 2E). Based on DSC measurements, the HEAT SENS LIPO sample has two phase transitions, one at 38.55 °C (onset) with 0.01 J/g enthalpy change, and another one at 41.46 °C (onset) with 0.23 J/g enthalpy change (Figure 2E). The inflection point of the drug release curve coincides with the first phase transition, which indicates that the release of the drug is not connected to the main chain-melting transition of the lipid bilayer.

#### 3.1.5. Stability of Liposomal Preparations

The HEAT SENS LIPO sample was subjected to a stability test. The sample was stored at 4 °C, and the ratio of free to liposome-bound copper-neocuproine complex was determined at pre-defined time points while using gel-filtration and UV-Vis spectroscopy. The results shown in Figure 2F indicate that the liposomes are stable for at least half a year. After 10 months, 80% of the active ingredient is still present in the liposome. Liposomes were also stable at 37 °C, with no drug release after four hours (liposomal drug content of 98%). Finally, the stability of the liposomes was also confirmed in completed medium (DMEM + 10% FBS) used to culture the cells (Figure S3).

### 3.2. Cytotoxic Effect of the Liposomes on Colorectal Cancer Cells

PrestoBlue assay was used to evaluate the in vitro cytotoxic effect of neocuproine, neucuproine-copper complexes, and their liposomal forms (HEAT RES LIPO and HEAT SENS LIPO) on C26 and HT-29 cells (Table 3). The toxicity of the samples was assessed at three different time points (4, 24, and 72 h) in order to determine the anticancer potential of the liposomal formulations, the active drug neocuproine and the different mixtures of neocuproine-copper(II) ions. Liposomal formulations were tested with and without pre-heating to 42 °C to model the added benefit of tumor mild hyperthermia treatments. In line with the stability of the liposomes, both liposomal formulations showed minimal toxicity without pre-heating. In contrast, after heating the liposomes to 42 °C, HEAT SENS LIPO was twice as effective as HEAT RES LIPO in the short-term (4 h) assay. In contrast, the toxicity of the heat-sensitive and heat-resistant formulations was identical after 24 h and 72 h incubation with cells, irrespective of the heat pretreatments. At 24 h, both liposomal formulations were more toxic than neocuproine, and less toxic than neocuproine-copper complexes. The toxicity of the combined copper and neocuproine treatment is due to the copper excess (2 µM Cu(II)) added to neocuproine. This is in line with earlier findings showing a considerable increase in the toxicity of intracellular copper ionophores in the presence of free copper ions [15]. We found that the toxicity of copper(II)-neocuproine complexes (1:1 or 1:2 ratio) is similar to that of the liposomal forms (although the 1:2 preformed complex was less effective in the 24-h experiment). Of note, the observation, that a chelator cannot be in excess if a rapid effect is required, is in line with earlier publications [18]. An excess of copper(II) ions in both the short and the long term resulted in lower IC_50_ values indicating a greater antiproliferative effect. In the longer, 72-h experiment, all investigated formulations showed comparable IC_50_ values against HT-29 and C26 cells.

### 3.3. Cu Accumulation by the Liposomal Formulation In Vitro

**Figure 3:**
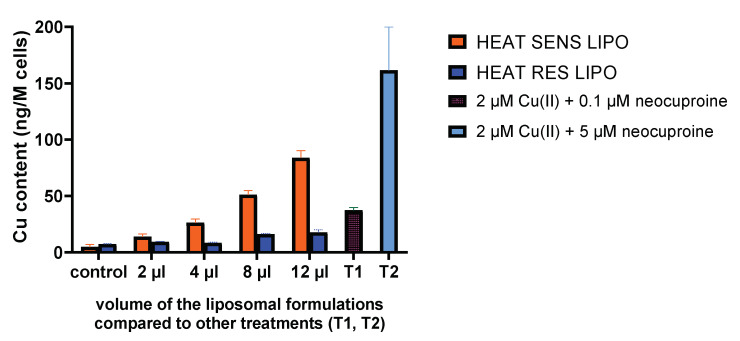
Cu content (measured by total-reflection X-ray fluorescence (TXRF) method) of C26 cell line treated for 4 h by different volumes of the liposomal formulations (HEAT SENS LIPO and HEAT RES LIPO) compared with the treatment of 0.1 μM (**T1**) and 5 μM neocuproine (**T2**) in the presence of 2 μM Cu(II) ions.

Copper accumulation was followed by TXRF to investigate the molecular background of in vitro toxicity. Treatment with increasing volumes of liposomes (HEAT RES LIPO and HEAT SENS LIPO) resulted in increasing copper content in C26 cells (Figure 3). Clearly, copper uptake mediated by HEAT SENS LIPO was much greater in this time range (4 h), as compared to the copper uptake mediated by HEAT RES LIPO, in agreement with the IC_50_ values that were measured at 24 h. Since tests modeling cell culture conditions did not indicate a difference in stability (see Section 3.1.5), we conclude that HEAT SENS LIPO accumulates copper better than the HEAT RES LIPO without using heat treatment. It should be noted that the highest copper uptake was observed following treatment with neocuproine in the presence of free copper [18].

### 3.4. In Vivo Effect of the Liposomal Formulations

**Figure 4:**
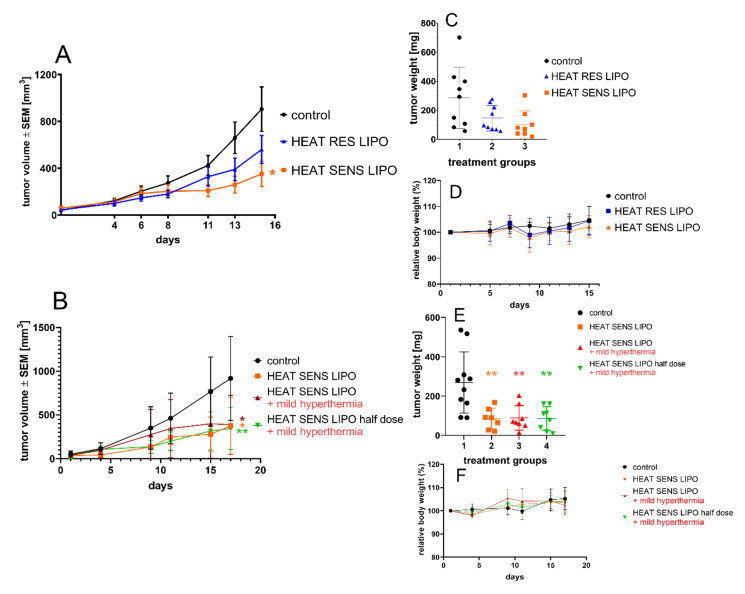
Antitumor efficacy of HEAT RES LIPO and HEAT SENS LIPO on C26 tumors in BALB/c animal model. (**A**) Average tumor volume and (**C**) dry tumor weight of vehicle, 2.8 mg/kg HEAT SENS LIPO and 2.5 mg/kg HEAT RES LIPO treated mice. Treatment groups received 10 µL liposome/1 g body weight or physiological saline solution intravenously (i.v.) on the 1st and 8th day of the treatment. (**B**) Average tumor volume and (**E**) dry tumor weight of vehicle, 2.8 mg/kg HEAT SENS LIPO, 2.8 mg/kg HEAT SENS LIPO + mild hyperthermia, 1.4 mg/kg HEAT SENS LIPO + mild hyperthermia mice. The treatments were implemented on the 1st and 10th day of the in vivo experiment. The local mild heat treatment was given immediately following drug injection (41–42 °C, 20 min). All data (**A**,**B**) presented as average ± SEM; * *p* < 0.05, ** *p* < 0.02. (**D**,**F**) Relative body weight data of animals during experiments.

The in vivo antitumor activity of the liposomal formulations (HEAT SENS LIPO and HEAT RES LIPO pegylated liposomes subjected to the appropriate formulation for mild and nonhyperthermic treatments, respectively) was tested in BALB/c mice bearing C26 tumors. In agreement with literature data [25], we found that the 1 mg/kg neocuproine-copper(II) complex is well tolerated, but it has no in vivo antitumor effect. Dosing of the liposomal formulations was designed to achieve maximum dose of neocuproine-copper(II) complex (2.8 mg/kg), which was tolerable for the animals. In the first experiment, the efficacy of HEAT SENS LIPO and HEAT RES LIPO was compared without applying heat treatments. Treatments with the liposomes showed a profound antitumor effect, reaching significance (*p* = 0.046) in the HEAT SENS LIPO group (Figure 4A). The antitumor effect was detectable in the weight of the dried tumor in both treated groups (Figure 4C) (65% and 50% tumor mass reduction in the HEAT SENS LIPO and HEAT RES LIPO groups, respectively). In the second experiment, our aim was to investigate whether using a smaller dose of thermosensitive liposome formulation combined with local heating of the tumor could show appropriate antitumor effect. Hence, the thermosensitive liposome formulation (HEAT SENS LIPO) was administered parallel with mild heat treatment in two doses (2.8 and 1.4 mg/kg) and compared to the group receiving higher dose treatment without mild heating (Figure 4B). The mild hyperthermia by itself showed no antitumor effect (data not shown). All three treated groups showed reduced tumor growth when compared to the control group (Figure 4B). Surprisingly, the strongest antitumor effect was observed as a result of treatment with 1.4 mg/kg liposome combined with mild heat treatment (*p* = 0.0145) and without heat (HEAT SENS LIPO) treatment (*p* = 0.0376) groups. At the beginning of the treatment, the “HEAT SENS LIPO + mild hyperthermia” group appeared to be less responsive to the treatment, but, over time, the effect leveled off (*p* = 0.0382). Referring to time-dependent in vitro experiments (Table 3), it should be noted that IC_50_ values of the cells that were treated with heated and non-heated liposomes become similar over time (72 h). The dried tumor masses (Figure 4E) in all groups were significantly lower (*p* values in the range of 0.0132–0.0153) than in the control group. Curves for each individual animals’ tumor volume is presented in Supplementary Figure S4. No decreases in body weight were observed during the experiments in mice receiving any liposomal treatments (Figure 4D,F).

### 3.5. PET/CT Results

**Figure 5:**
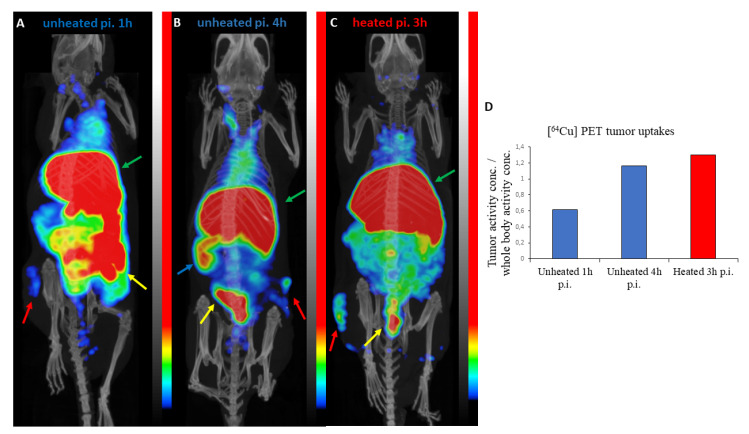
Positron emission tomography/CT (PET/CT) imaging results. Maximum intensity projections of PET/CT images showing the biodistribution of [^64^Cu]Cu-neocuproine liposomes (HEAT SENS LIPO) in control (**A**,**B**) and mild hyperthermia treated (**C**) mice. The liver (green arrows) and intestine (yellow arrows) of all animals show high activities. The tumor (red arrows) also shows increased uptake and is easily distinguishable from its surroundings on all images. The spleen (blue arrow) of a control animal shows high uptake 4 h p.i. (**B**). There is a marked difference between the tumor activity concentrations (normalized to whole body concentrations) between the 1 h p.i. and 4 h p.i. control animals (**D**).

The in vivo distribution of [^64^Cu]Cu-neocuproine liposomes (HEAT SENS LIPO) was evaluated in a U251-HRE mouse xenograft model (*n* = 3). Figure 5 shows representative PET/CT images. The tumor activity concentration/whole body activity concentration ratios were 0.61 and 1.16 for the control animals (1 h and 4 h p.i., respectively) and 1.30 for the animal receiving mild heat treatment (3 h p.i.). High activity concentrations were observed in the livers and intestines of all three mice and the spleen of one of the control animals 4 h post injection. This uptake pattern in the organs has been observed with other liposomal drug delivery systems [48,49]. Although biodistribution is greatly influenced by the size, lipid composition, and surface modification of liposomes [50,51], it is likely that the high intestinal activity observed in these animals is caused by the hepatic clearance of free ^64^Cu ions [49,52,53] or [^64^Cu]Cu-neocuproine released from the liposomes. The tumor-to-muscle ratios (representing the target-to-background ratio) were 4.47 and 9.70 for the control animals (p.i. 1 h and p.i. 4 h, respectively) and 4.64 for the heat treated animal indicating good target visibility (a ratio of at least 1.5 is necessary for the identification of a lesion [54]). The imaging results suggest efficient tumoral drug uptake; however, further studies are required to accurately determine the effect of local heating of the tumor on the amount of free or neocuproine-bound copper released in the circulation.

## 4. Conclusions

In this study, we demonstrated the feasibility of using a liposome preparation containing a copper nanotoxin and excess of copper ion in a water-soluble form for tumor targeting. In addition, the preparation of a thermosensitive pegylated liposome is presented by the proper use of DPPC/HSPC lipid ratio. The thermosensitive and the “thermoresistant” formulations both induce intracellular copper accumulation and related in vitro cytotoxic effects. Even without pre-heating, the activity of the HEAT SENS LIPO formulation was superior both in terms of cellular metal accumulation and in vitro toxicity. In vivo antitumor activity was detectable with both liposomes, but the effect of HEAT SENS LIPO is more pronounced. Mild hyperthermia treatment, combined with the HEAT SENS LIPO formulation, allowed for the reduction of the applied dose. Taken together, our results demonstrate the feasibility of targeting a copper nanotoxin encapsulated in thermosensitive liposomes containing an excess of copper.

## Figures and Tables

**Table 1:** Lipid composition of the prepared liposomes in weight ratios. All liposomes are drug loaded.

Sample Name	DPPC (mg)	HSPC (mg)	DSPE-PEG2000 (mg)	*w*/*w*% of DPPC and HSPC
LIPO1 hereinafter **HEAT RES LIPO**	0	30	5	100%HSPC:PEG
LIPO2	15	15	5	50%DPPC:50%HSPC:PEG
LIPO3 hereinafter **HEAT SENS LIPO**	21	9	5	70%DPPC:30%HSPC:PEG
LIPO4	24	6	5	80%DPPC:20%HSPC:PEG
LIPO5	27	3	5	90%DPPC:10%HSPC:PEG
LIPO6	30	0	5	100%DPPC:PEG

**Table 2:** In vitro data (IC_50_) using PrestoBlue assay on HT-29 cell line in a four-hour experiment. Phase transition temperature optimization of the liposomal formulation using the different *w*/*w*% 1,2-dipalmitoyl-sn-glycero-3-phosphatidylcholine (DPPC) and hydrogenated soybean phosphatidylcholine (HSPC) mixtures (Table 1) for using heat treatment.

Liposome Formulation	IC_50_ (µM) (RSD < 20%)		
	37 °C	38 °C	39 °C
LIPO1 HEAT RES LIPO	>40	>40	~40
LIPO2	>40	>40	~40
LIPO3 HEAT SENS LIPO	>40	>40	36.8
LIPO4	>40	30.9	15.5
LIPO5	>40	9.9	5.2
LIPO6	~40	5.9	4.6

**Table 3:** Time dependent (4 h, 24 h, and 72 h) IC_50_ values (RSD < 20%) on HT-29 and C26 cells of the prepared liposomes (HEAT SENS LIPO and HEAT RES LIPO) at different temperatures as compared to neocuproine chelator, to copper-neocuproine preformed complexes (different ratios: 1:1 and 1:2) and to neocuproine chelator in the presence of 2 µM copper(II) ions.

Treatment Parameters		IC_50_ Values (µM) on HT-29 and C26 Cell Lines (RSD < 20%)							
		HEAT SENS LIPO		HEAT RES LIPO		Compared to other Treatments			
Cell Line	Time	Without Pre-Heating 37 °C	Heating to 42 °C	without Pre-Heating 37 °C	Heating to 42 °C	Neocuproine	2 µM Cu(II) + Neocuproine	Cu(II)-Neocuproine Preformed Complex 1:1	Cu(II)-Neocuproine Preformed Complex 1:2
HT-29	4 h	>40	19.4	>40	>40	>500	1.6	1.15	n.a.
	24 h	5	2.8	9.8	10.1	~300	0.16	0.6	>25
	72 h	0.2	0.2	0.2	0.3	0.083	0.025	0.05	0.053
C26	4 h	>40	18.6	>40	>40	>500	1.8	1.05	n.a.
	24 h	3.6	3	4.2	4.1	~500	0.21	0.49	>25
	72 h	0.2	0.15	0.3	0.2	0.15	0.037	0.13	0.12

## Supplementary Materials

The following are available online at https://www.mdpi.com/1999-4923/12/5/466/s1, Figure S1: Final purification of Cu-64 labelled HEAT SENS LIPO on a PD-10 column, Figure S2: Optimization of the mol lipid: mol drug ratio in the case of HEAT SENS LIPO, Figure S3: SEC method combined with UV/Vis-spectrophotometry was used to check the integrity of the liposomes in completed media (containing 10% FBS), Figure S4: Growth curves of individual tumors (from Figure 4B), Table S1: Optimization of the copper(II) concentration for the preparation of thermosensitive liposomal formulation, Table S2: Optimization of drug to phospholipid ratio during the liposomal formulation process, Table S3: In vitro IC50 values (µM) of liposomes with different compositions.
