# Supplementary material for: Development and In Vivo Application of a Water-Soluble Anticancer Copper Ionophore System Using a Temperature-Sensitive Liposome Formulation
Source: Pharmaceutics. 2020 May 20;12(5):466. doi: 10.3390/pharmaceutics12050466 (PMC7284829; doi:10.3390/pharmaceutics12050466)
Supplement: Supplementary file 1 [file pharmaceutics-12-00466-s001.pdf]

pharmaceutics

Supplementary Materials: Development and In Vivo Application of a Water-Soluble Anticancer Copper Ionophore System Using a Temperature-Sensitive Liposome Formulation

Anikó Gaál, Tamás M. Garay, Ildikó Horváth, Domokos Máthé, Dávid Szöllősi, Dániel S. Veres, Jeremiah Mbuotidem, Tibor Kovács, József Tóvári, Ralf Bergmann, Christina Streli, Gergely Szakács, Judith Mihály, Zoltán Varga and Norbert Szoboszlai

Table S1.: Cu concentration, IC_{50} values and DLS parameters of LIPO1 (in the case of 100mM $CuSO_4$ referred as HEAT RES LIPO) for the optimization of copper(II) concentration for the preparation of thermosensitive liposomal formulation. 300 mM $CuSO_4$ loading resulted in increased PDI index according to DLS results, while the lowest concentration of 10 mM $CuSO_4$ loading resulted in significantly lower Cu concentration (as measured after gel filtration with TXRF method) and markedly decreased in vitro cytotoxic effect (1day experiment using PrestoBlue assay).

LIPO1				
**Concentration**	**Cu (mM)**	**IC_{50} (μM) 24 h (RSD < 20%)**	**D_{avr} ± SD (nm)**	**PDI (%)**
10 mM $CuSO_4$	0.5 ± 0.1	25.8	94 ± 12	13
100 mM $CuSO_4$	1.0 ± 0.2	3.3	103 ± 13	12
300 mM $CuSO_4$	2.5 ± 0.3	2.8	120 ± 24	20

Table S2. Optimization of drug to phospholipid ratio during the liposomal formulation process. The optimal drug-to-lipid concentration was investigated in the 0.2 mol–0.8 mol neocuproine to 1 mol lipid concentration range. 0.2 mol of neocuproine per 1 mol of phospholipid was selected for further experiments. IC50 value was a good selection parameter: The difference between 0.1 mol and 0.2 mol neocuproine amount (to 1 mol phospholipid content) is still significant but does not change substantially at higher neocuproine mol ratio, however, the polydispersity of the liposome increase in the case of the lipid composition corresponding to the HEAT SENS LIPO formulation.

**Liposomal Formulation**	**IC_{50} (μM) 24 h (RSD < 20%)**	**D_{avr} ± SD (nm)**	**PDI (%)**
HEAT SENS LIPO 1mol lipid:**0.1 mol neocuproine** ratio	5.9	92 ± 10	10
HEAT SENS LIPO 1mol lipid:**0.2 mol neocuproine** ratio	3.5	97 ± 8	9
HEAT SENS LIPO 1mol lipid:**0.4 mol neocuproine** ratio	3.2	98 ± 14	16
HEAT SENS LIPO 1mol lipid:**0.8 mol neocuproine** ratio	4.1	95 ± 13	15
HEAT RES LIPO 1mol lipid:**0.1 mol neocuproine** ratio	9.1	104 ± 13	14
HEAT RES LIPO 1mol lipid:**0.2 mol neocuproine** ratio	4.2	103 ± 13	12
HEAT RES LIPO 1mol lipid:**0.4 mol neocuproine** ratio	3.9	102 ± 12	14
HEAT RES LIPO 1mol lipid:**0.8 mol neocuproine** ratio	4.8	103 ± 12	14

Table S3. In vitro IC50 values (μM) of drug loaded liposomes (LIPO1-LIPO6) with different compositions and **Cu(II)-neocuproine preformed complex 1:1**. Prestoblue assay on HT-29 cells were applied to identify IC50 values in 4h and 24h treatment with intact and with preheated (10 min, 45 °C) liposomes. No significant difference in IC50 values (μM) was observed between the different lipid compositions regardless of pretreatment and duration of the assay, but the liposome containing 100% HSPC was the least effective.

**Different Liposomal Formulations**	**Lipid Composition**		**IC50 (μM) 4 h (RSD < 10%)**		**IC50 (μM) 24 h (RSD < 20%)**	
	**DPPC** *w/w%*	**HSPC** *w/w%*	**without previous heating**	**heating to 45 °C for 10 min before the experiment**	**without previous heating**	**heating to 45 °C for 10 min before the experiment**
LIPO1 HEAT RES LIPO	0	100	33.1	27.5	4.3	2.6
LIPO2	50	50	> 40	10.1	2.1	0.98
LIPO3 HEAT SENS LIPO	70	30	> 40	9.2	2.2	0.94
LIPO4	80	20	> 40	6.5	2.0	0.91
LIPO5	90	10	> 40	5.5	1.5	0.62
LIPO6	100	0	> 40	5.2	1.7	0.71
Cu(II)-neocuproine preformed complex 1:1	-	-	1.15	n.a.	0.6	n.a.

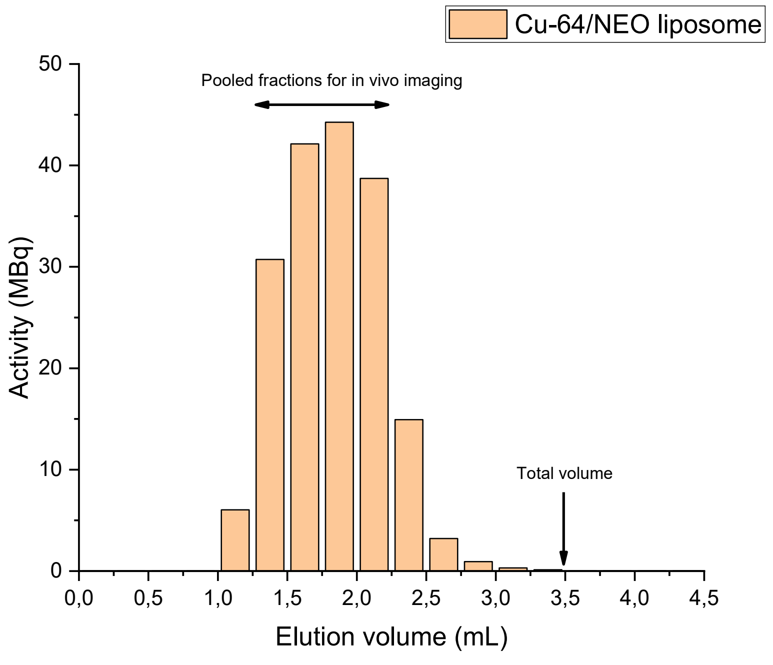

Figure S1. Final purification of Cu-64 labelled HEAT SENS LIPO on a PD-10 column.

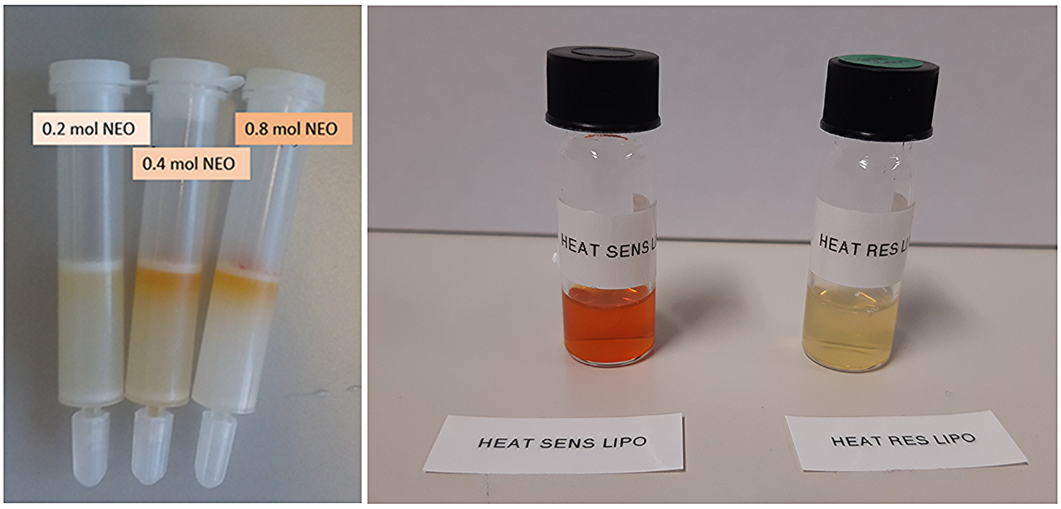

Figure S2. Optimization of the mol lipid: mol drug ratio in the case of HEAT SENS LIPO. Increasing the amount of the neocuproine chelator does not result an increased in vitro cytotoxic effect (Table S2.), because it increase the amount of the precipitated complex in the external buffer system. This remains on the column in the final purification step with an orange color corresponding to the color of the Cu(I)-neocuproine complex (left). The resulted liposomal formulations: HEAT SENS LIPO and HEAT RES LIPO are shown on the right figure.

Sample	Sec Chromatogram
**HEAT SENS LIPO**	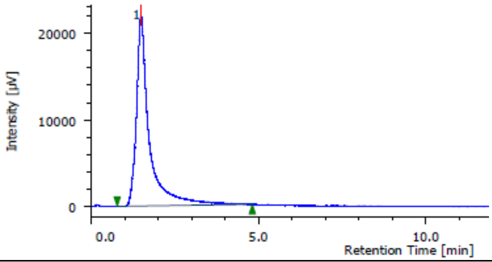
**HEAT SENS LIPO incubated in completed DMEM for 4 h at 37 °C, 5% CO_2**	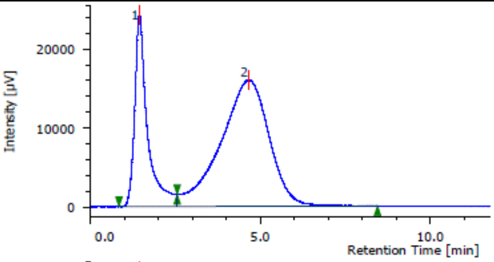
**HEAT RES LIPO**	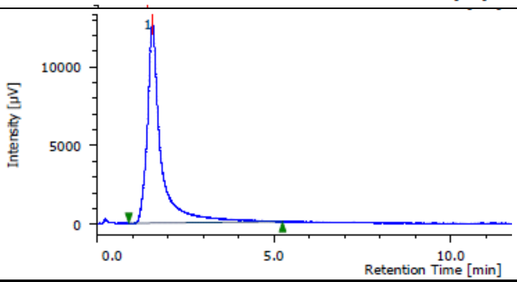

**HEAT RES LIPO incubated in completed DMEM for 4 h at 37 °C, 5% CO_2**	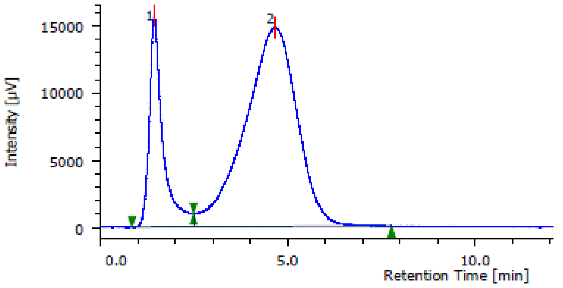
**Completed DMEM**	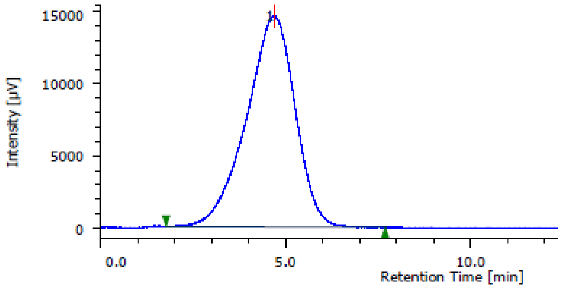

Figure S3. SEC chromatograms for stability investigation. SEC method combined with UV/Vis-spectrophotometry was used to check the integrity of the liposomes in completed media (containing 10% FBS). SEC chromatograms indicate that the active ingredient was not released by the liposomes in the presence of completed cell culture media.

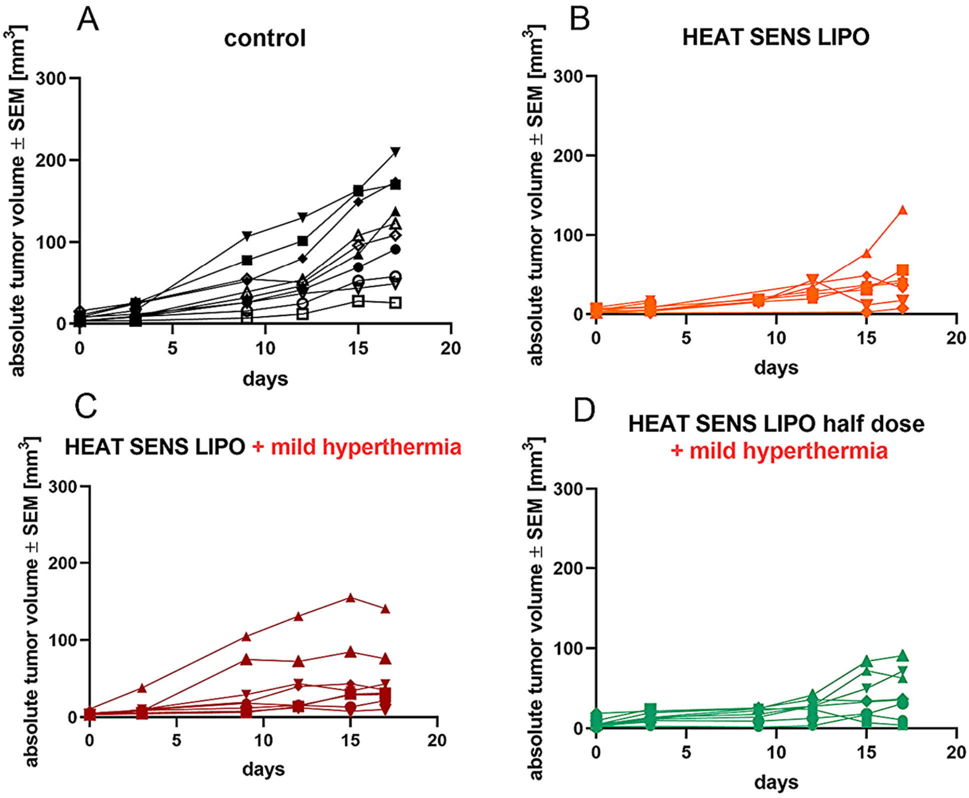

Figure S4. Growth curves of individual tumors (from Figure 4B) in control (**A**), HEAT SENS LIPO (**B**), HEAT SENS LIPO + hyperthermia (**C**), half dose of HEAT SENS LIPO + hyperthermia treated mice (**D**).
